# Effect of pine essential oil and rotating magnetic field on antimicrobial performance

**DOI:** 10.1038/s41598-022-13908-5

**Published:** 2022-06-11

**Authors:** Agata Markowska-Szczupak, Aneta Wesołowska, Tomasz Borowski, Dawid Sołoducha, Oliwia Paszkiewicz, Marian Kordas, Rafał Rakoczy

**Affiliations:** 1 Department of Chemical and Process Engineering, Faculty of Chemical Technology and Engineering, West Pomeranian University of Technology in Szczecin, Piastów Ave. 42, 71-065 Szczecin, Poland; 2 Department of Organic and Physical Chemistry, Faculty of Chemical Technology and Engineering, West Pomeranian University of Technology in Szczecin, Piastów Ave. 42, 71-065 Szczecin, Poland

**Keywords:** Chemical engineering, Biochemistry

## Abstract

This work presents the results ofa study which concerns the influence of rotating magnetic field (RMF) on the antibacterial performance of commercial pine essential oil. A suspension of essential oil in saline solution and *Escherichia coli* were exposed to the rotating magnetic Afield (the frequency of electrical current supplied by a RMF generator f = 1–50 Hz; the averaged values of magnetic induction in the cross-section of the RMF generator B = 13.13 to − 19.92 mT, time of exposure t = 160 min, temperature of incubation 37 °C). The chemical composition of pine (*Pinus sylvestris* L.) essential oil was determined by gas chromatography coupled with mass spectrometry (GC–MS). The main constituents were α-pinene (28.58%), β-pinene (17.79%), δ-3-carene (14.17%) and limonene (11.58%). The present study indicates the exposition to the RMF, as compared to the unexposed controls causing an increase in the efficacy of antibacterial properties of pine oil. We have shown that rotating magnetic fields (RMF) at a frequency, f, between 25 Hz to and 50 Hz increased the antimicrobial efficiency of oil a concentration lower than 50%.

## Introduction

In recent years the use of varied magnetic therapy for treating pain, chronic wounds (magnetotherapy), infectious diseases (e.g. malaria), allergies or even cancer is gaining in popularity ^1–4^. The applied magnetic field has a frequency below 100 Hz and magnetic flux density in the range between 0.1 and 30 mT ^3^. It is a well-known method of non-thermal food preservation and water disinfection ^5^. However, the mechanism of an electromagnetic field in killing microorganisms is not fully explained and the obtained results are contradictory and confusing ^6,7^. The positive effect of magnetic field was observed by Junka et al. as a decrease of *Staphylococcus aureus* and *Pseudomonas aeruginosa* bio-film formation ^8^. The proposed mechanisms of antibacterial impact of electromagnetic fields involved: (*i*) increase in the permeability of ionic channels in the membrane, (*ii*) disruption of nutrient and ion transport (e.g. K^+^), (*iii*) formation of free radicals due to magnetic field exposure, (iv) increased oxygenation and temperature rise due to increased particle movement or (v) mechanical damage to cell walls ^1^. On the contrary, according to Fijałkowski et al. exposure to the rotating magnetic field (RMF) of B = 34 mT (f = 50 Hz) increased the growth rate, proliferation, cell metabolic activity and biofilm of *Staphylococcus aureus, Escherichia coli, S. marcescens*, *S. mutans*, *Cronobacter sakazakii*, *Klebsiella. oxytoca* and *S. xylosus* formation but had a negative impact on *Acinetobacter baumannii* and *P. aeruginosa.* Moreover, in a distinct line of investigation, it was demonstrated that RMF—induced bacterial growth and could be used for the production of bacterial cellulose (BC) ^9^. This biomaterial displays more favorable properties when RMF is applied than in non-RMF induced conditions ^10,11^. It was also proved that the magnetic field impact depends on its type, magnetic induction and frequency. A possible explanation of this phenomenon is the exertion of cell transport or the mixing rate of bioliquids and transfer between the cell surface increased ^11^. Although many authors reported varied effects of the magnetic field, its applications are still missing. It gives us motivation for continuing research on this issue. Another inspiration was given by Yadav et al. ^12^. We decided to investigate the influence of rotating magnetic field (RMF) generated in generated in a reactor originally constructed by our team on the antimicrobial activity of pine essential oil. Similar concentrations of the tested oil were used as well as the same model the Gram-negative bacteria (*Escherichia coli*). The magnetic fields with frequencies ranging from 5 to 50 Hz were applied. A commercial pine essential oil (EO), which is known for its strong aroma was selected for tests. This oil is widely used in aromatherapy, cosmetic, pharmaceutical and packing industries. Pine oil offers multiple advantages such as low price and toxicity (it is one of the safest essential oil) and good blending properties (it blends well with many other oils including cedarwood, rosemary, lavender, sage, labdanum, and juniper) ^13^. However, it showed moderate antimicrobial activity against *E. coli* and other pathogenic bacteria ^14^. The main goal was to examine if the synergistic effect of RMF and pine oil is achievable.

## Results and discussion

Due to its continued use in aromatherapy pine is one of the most popular plants throughout all civilization. Pine oil is highly concentrated in needles, twigs, and buds which are easily obtained ^15^. Pine essential oils are volatile constituents (a complex mixture of polar and non‐polar compounds) obtained from needles of the pine tree. The composition strongly depends on the species (or genus) of the extracted plant, the geographic location of this plant, degree of needle pollution, harvest time and extraction techniques ^14,16^. As shown in Supplementary Materials SM 1, forty-four components were identified in the tested pine essential oil, representing 99.87% of the total oil. Based on the analysis of GC–MS chromatogram (Supplementary Materials SM 2) of pine essential oil, the major constituents were α-pinene (28.58%), β-pinene (17.79%), δ-3-carene (14.17%) and limonene (11.58%). Oxygenated monoterpenes and sesquiterpene hydrocarbons were present in amounts less than 10% (Table 1). In other research on *Pinus heldreichii*, *P. peuce*, *P. mugo*, *Pinus nigra* and *P. sylvestris* essential oils as many as 112 compounds were identified ^15^. On the other hand, in oil from needles (EON) of cembran pine, only 27 components were detected ^17^. In general, the tested pine oils were customarily characterized by the type of compound that was most prevalent in T them: α-pinene and β-pinene, limonene, phellandrene and cadinene. The other components found in significant amounts were caryophyllene (5.18%), terpinolene (4.62%) and bornyl acetate (4.55%). Monoterpene hydrocarbons (84.80%) were found to be the dominant subclass of identified components (Table 1).

**Table 1:** Chemical composition of pine essential oil.

Class and subclass of compounds	%
**Monoterpenoids**	91.19
Monoterpene hydrocarbons (MH)	84.80
Oxygenated monoterpenes (OM)	6.39
**Sesquiterpenoids**	8.37
Sesquiterpene hydrocarbons (SH)	8.08
Oxygenated sesquiterpenes (OS)	0.29
**Others (O)**	0.31
**Total identified**	99.87

As shown by Maciąg et al., despite fcomposition of *Pinus sylvestris* essential oil from different areas of Europe the most common group of volatile compounds are monoterpene hydrocarbons, followed by sesquiterpene hydrocarbons and oxygenated sesquiterpene ^18^. It is well known that the composition of essential oils and the contents of toxic elements determine the antimicrobial activity of pine oils ^13,14^.

The sensitivity of commercial pine essential oil against *Escherichia coli* by disc diffusion assay showed a significant inhibition of *E. coli* growth when pine essential oil was used at high concentrations (100% and 50%). This finding is consistent with other research studies on the topic ^18,19^. There were no zones of growth inhibition around discs soaked with pine essential oil in concentration from 6.25 to 25% and 0.85.% NaCl buffer (control). The results are presented in Supplementary Materials SM 3. Research on how different essential oils affect the growth of pathogenic bacteria revealed that pine oil was not the most effective oil ^18^. No activity against *E. coli* was even confirmed in a certain study ^20^.

The minimum concentration of commercial pine oil against *E. coli* supported the results of disc diffusion assay. The maximum antimicrobial activity for oil suspensions in the range from 50.0 to 100.0% (after 160–180 min) was obtained (after 60–120 min).

**Figure 1:**
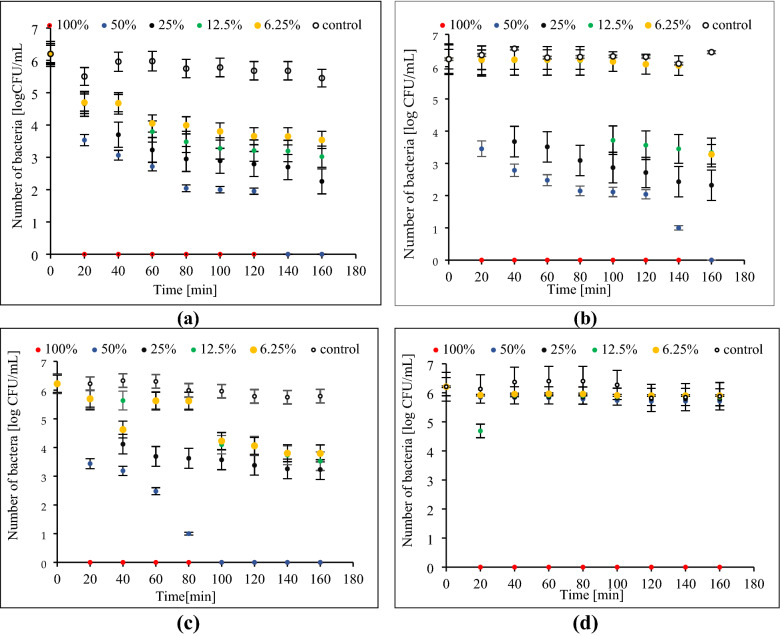
Antibacterial potential of pine oil against *Escherichia coli* at rotating magnetic field (RMF) (**a**) 5 Hz; (**b**) 25 Hz; (**c**) 50 Hz, (**d**) non-electromagnetic field.

In Fig. 1 antibacterial effectiveness of pine oil against *Escherichia coli* at rotating magnetic field (RMF) of varied frequencies of 5, 25 and 50 Hz and in non-electromagnetic field conditions is presented.

It was shown that 100% concentration of pine oil resulted in a total reduction of bacterial number regardless of the applied RMF frequency as well as in non-electromagnetic field conditions after 20 min of the process (Fig. 1a–d). In accordance with the outcomes of MIC results, 50% concentration of pine oil led to the same effect after 160 min when the frequency of RMF was 5 and 25 Hz, and 100 min when the frequency was 50 Hz. (Fig. 1a–c). There was a significant decrease in bacterial number, regardless of pine oil concentration (Fig. 1b).

The RMF effect on the antibacterial potential of pine oil against *E. coli* might be expressed in the following form:

1 $$N^{*} = f(c,t) \Rightarrow \left\{ {\frac{{N|_{{f = {\text{var}} }} }}{{N_{0} }}} \right\} = f(c,t)$$

where: N*—relative number of bacteria; *N*|_f=var_—number of bacteria in samples expressed at RMF (for the various frequencies of electrical current, f), log CFU·mL^-1^; *N*_0_—number of bacteria in control samples (without the application of RMF), log CFU·mL^-1^; c—concentration of pine essential oil, %v/v; t—time, min.

Response surface methodology (RSM), based on the design of experiments (DoE) is a set of statistical and mathematical tools for designing experiments and optimizing process parameters ^21,22^. This serves as a mathematical tool for optimization. The main aim of this method is to determine the interaction between independent variables, alone or in combination, in the form of a mathematical model ^23^. This model generates an accurate mathematical description of the overall process and may be used to analyze the effects of independent variables. The RSM method is based on the description of interactive effects of process variables in the form of graphical representation ^21^. To obtain the response surface and the second-order polynomial models in RMS the fractional designs (the Central Composite Design (CCD) or Box-Behnken Design (BBD)) were applied ^24^. The application of RSM allowed to select the best experimental conditions requiring the lowest number of experiments to obtain the appropriate results ^25,26^.

The RSM approach is a mathematical method for modeling and analyzing a process in which the response is affected by various variables. The RSM method is suitable for fitting a quadratic surface and it helps to optimize process parameters and to analyze the interaction between independent parameters in an experiment. In this study, two independent variables were chosen for the statistical experiment design as follows: concentration of pine essential oil and time. The relative number of bacteria was treated as the response of the system.

The main aim of this method is to optimize the response surface and to understand the topography of the response surface by taking into consideration the local minimum, local maximum or analyzing how this surface varies with the independent values. In RSM approach, response surface is a graphical representation used to describe the interactive effects of process variables and their effects on response (in this work it is the relative number of bacteria).

The RSM method allows to evaluate the experimental data with a (linear, quadratic, cubic or two-factor interaction) statistical model. The obtained results were analyzed with a using the second-order polynomial equation developed to fit the experimental data and a to identify the relevant model terms:

2 $$Y = \beta_{0} + \beta_{1} x_{1} + \beta_{2} x_{2} + \beta_{12} x_{1} x_{2} + \beta_{11} x_{1}^{2} + \beta_{22} x_{2}^{2} + \varepsilon$$

where: *Y—*the predicted response; *β*_*0*_—the constant coefficient; *β*_*1*_, *β*_*2*_—the linear coefficient; *β*_*11,*_* β*_*22*_—the quadratic coefficient; *β*_*12*_—the interaction coefficient; *x*_*i*,_ *x*_*j*_*—*the process variables; *ε*—the residual error.

The second order model (Eq. 2) was developed using Statistica 13.3 (TIBCO Software Inc.). This model includes linear terms, second order term for the process variables, and cross product terms. It is the basis for response surface designs under the assumption that although the hill is not a perfect quadratic polynomial in analyzed dimensions, it gives a good approximation to the surface near the maximum or the minimum. It should be emphasized that the goal of this investigation was to obtain the local minimum of the predicted response. To assess the effect of independent values (time and concentrations of pine essential oil) on the relative number of bacteria, the following relationship was applied:

3 $$N^*=p_1+p_2c+p_3c^2+p_4t+p_5t^2+p_6ct$$

where: p_1_–p_9_: parameters represent the intercept, linear, quadratic and interaction coefficients.

To find out the optimum levels of parameters for improving the proposed antimicrobial method, the RSM based on the CCD was used. The significance of the process parameters and their interactions was tested by means of ANOVA with a 95% confidence level. Overall model significance was determined according to Fisher’s F-test and its associated probability. The coefficient of determination (R^2^) was applied to evaluate the quality of the polynomial equation fit. A three-dimensional surface plot was applied to illustrate the relationship between response (relative number of bacteria) and the experimental levels of independent values.

**Figure 2:**
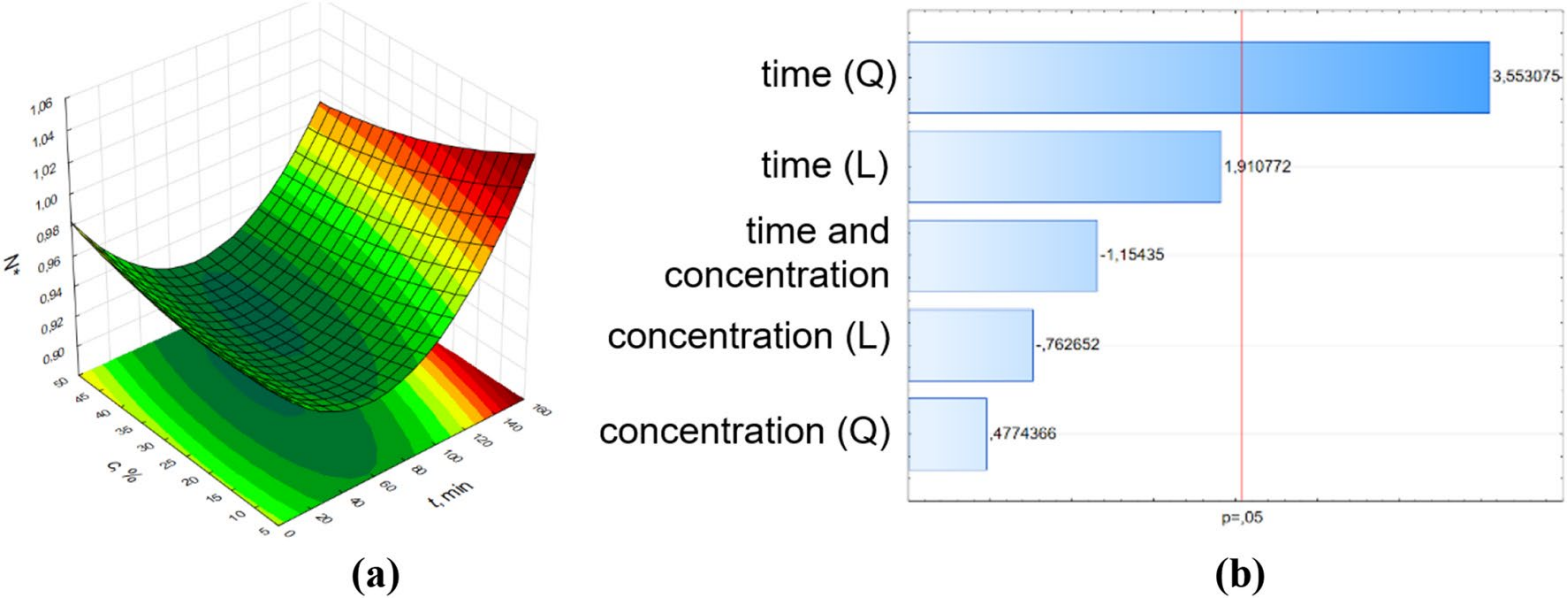
Surface response graphs of antibacterial potential (**a**) and Pareto chart (**b**) of pine oil against *E. coli* for control samples (without the influence of RMF).

**Figure 3:**
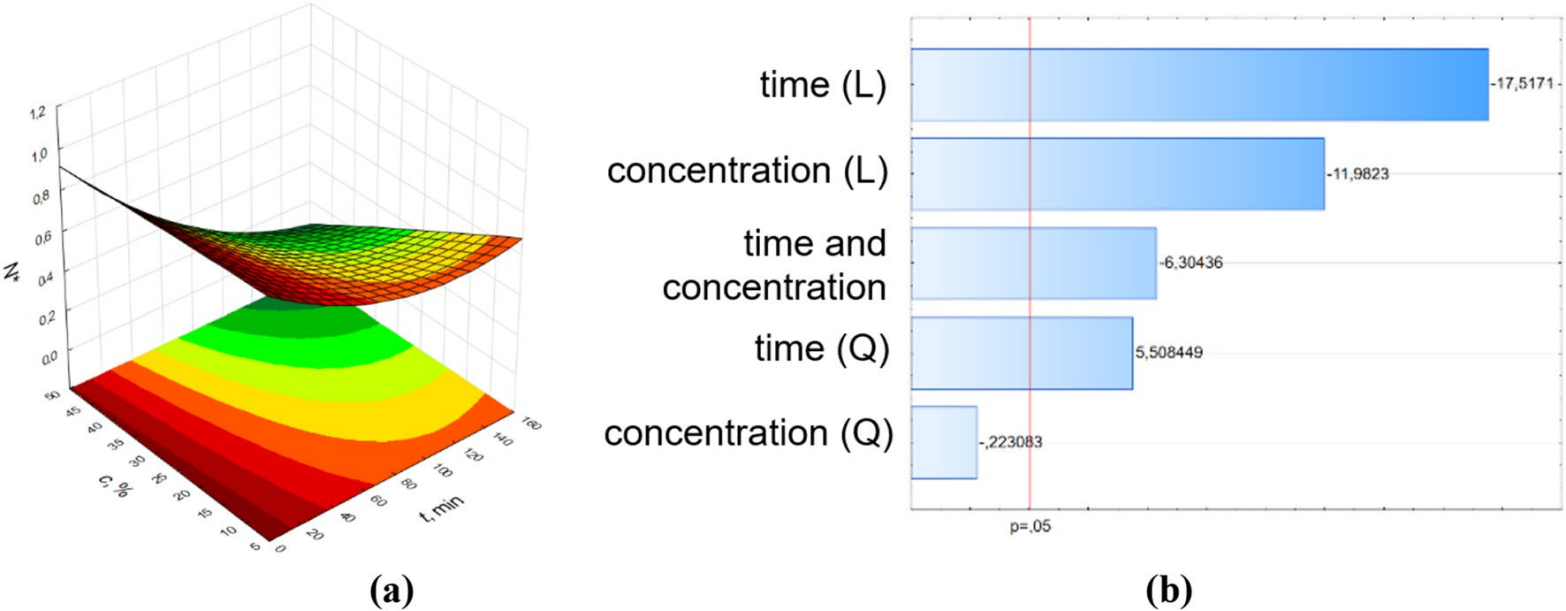
Surface response graphs of antibacterial potential (**a**) and Pareto chart (**b**) of pine oil against *E. coli* for samples with the effect of RMF (f = 5 Hz).

**Figure 4:**
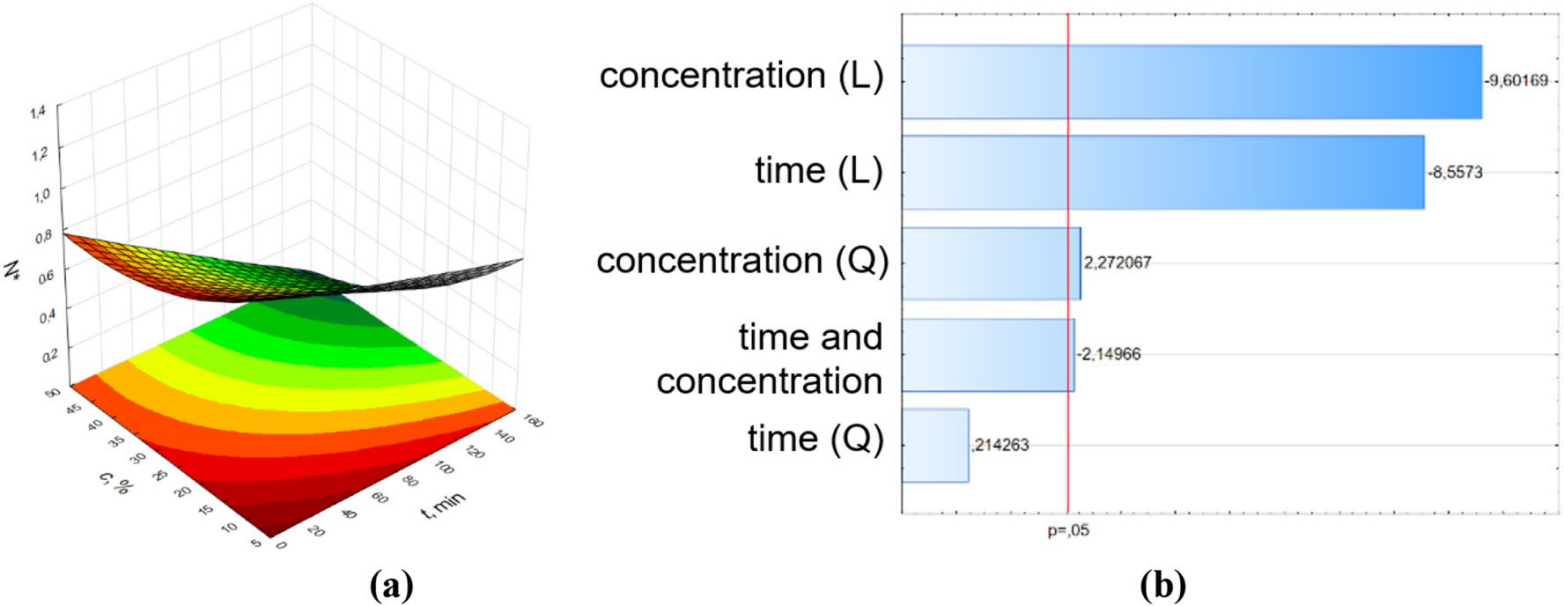
Surface response graphs of antibacterial potential (**a**) and Pareto chart (**b**) of pine oil against *E. coli* for samples with the effect of RMF (f = 25 Hz).

**Figure 5:**
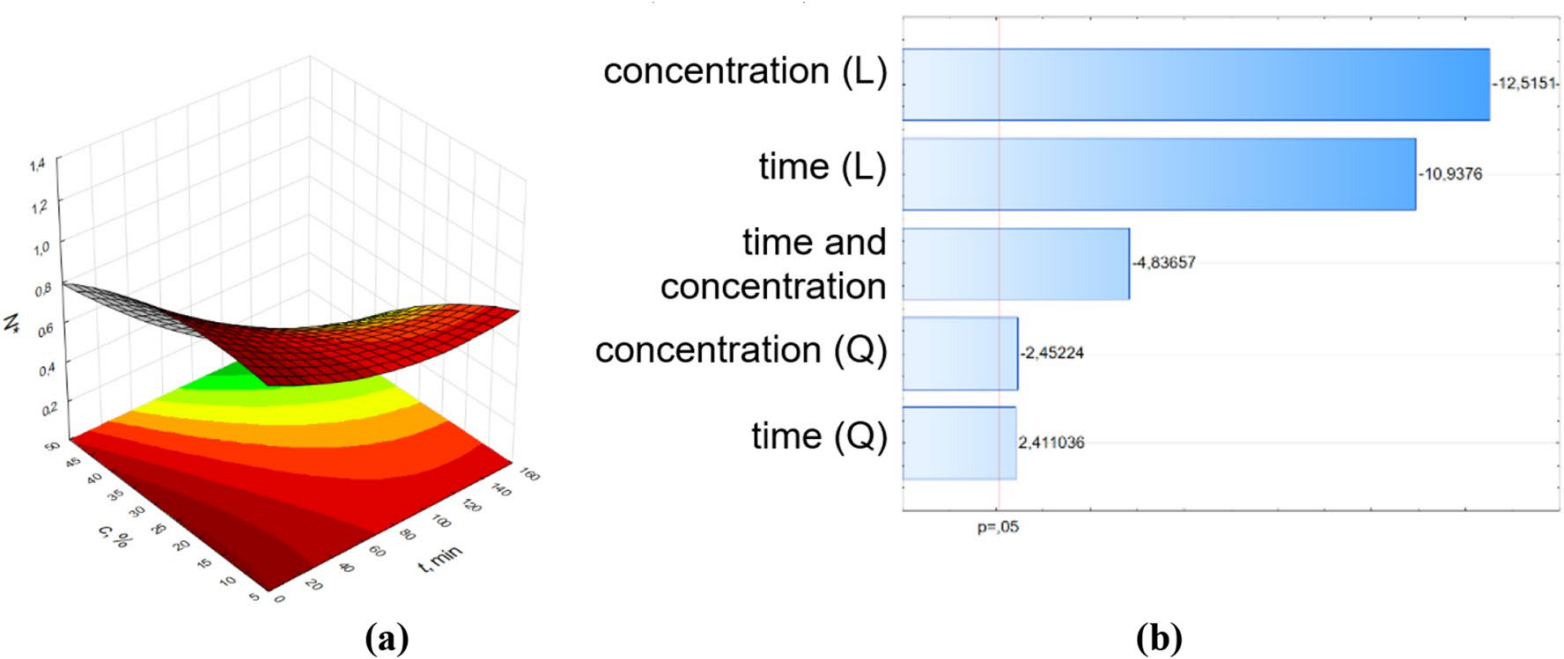
Surface response graphs of antibacterial potential (**a**) and Pareto chart (**b**) of pine oil against *E. coli* for samples with the effect of RMF (f = 50 Hz).

To detect the factor and interaction effects that are most important to the process, the Pareto plot was used ^27,28^. This plot clearly illustrates which effects (time or concentrations of pine essential oil) and two-factor interactions (between time and concentrations of pine essential oil) are statistically significant at 5% significance level. To obtain the optimal response, the interaction between the two variables (time and concentrations of pine essential oil) were visualized through the response surface graphs and Pareto charts (Figs. 2, 3, 4, 5).

The results of ANOVA analysis were complemented by Pareto analysis. Parteo charts were prepared to express the influence of the variables on the response. The most important variable is determined by selecting variables that show the greatest standardized effect to most responses in these graphs. The length of the bar is proportional to the standardized effect. The symbols *L* and *Q* denote the linear and quadratic effect in Eq. (3), respectively.

The parameters values of Eq. (2) and the coefficients of determination (R^2^) from the RSM method are presented in Supplementary Materials SM 4. In the Pareto charts, the parameters which cross the reference line at 0.05 represent the factors with the highest statistically significant influence. From Pareto chart analysis, significant effects were observed on the relative number of bacteria (number of bacteria related to the control sample) when varying pine oil concentration and time. Also, as shown in the Pareto chart for the control sample (Fig. 2b), only parameter t^2^ (squared time) has a significant effect on N^*^ values. In case of the influence of RMF on the antimicrobial potential of the applied pine oil, significant effects were obtained for almost all parameters of Eq. (1). The concentration of pine oil and time had no significant effects on the samples at 25 Hz and 50 Hz, respectively.

The obtained surface response graphs (Figs. 2a–5a) were plotted to show the mutual interactions between the independent variables. The graphs might be used to identify conditions under which the response is optimal. From the point of experimental strategy, the combinations of factors that optimize the response should be determined. Therefore, the results obtained through the surface graphs made it possible to establish the best response when the relative number of bacteria was analyzed as the response variable. From these plots, it was found the smallest N* occurred when both concentrations of pine oil and time were at their highest point. It should be noticed that the calculated values of N* from the proposed mathematical description for t = 160 min and c = 50% are equal to 0.99 for the control sample, 0.28 for 5 Hz, 0.17 for 25 Hz, and 0 for 50 Hz.

The RMS method might be used to investigate the individual and interaction effect of the operational parameters (concentration of pine essential oil and time) on the response (relative number of bacteria). Figures 2, 3, 4, 5 also demonstrate the interactions between the variables in three-dimensional response surface plots. Based on the obtained models, the response surfaces were derived and the Pareto ranking was carried out. This analysis indicated that the most important variable affecting the response was time for samples without the effect of RMF and with the effect of RMF (f = 5 Hz). The application of RMF at f = 25 Hz and f = 50 Hz increased the importance of the concentration of pine essential oil. The obtained mathematical models not only indicated the most relevant factors that influence the antibacterial potential of pine oil under the action of RMF but also make it possible to reduce RMF exposition time.

Via looking within the literature of pine oil antibacterial properties, it was summarized that monoterpene and sesquiterpene compounds in *Pinus* sp. essential oil are identified as the main compounds responsible for the bacteriostatic effect. The comparison of MIC values of α-pinene and β-pinene enantiomers indicates that positive enantiomers exhibited a microbicidal effect against microorganisms, with MIC values ranging from 117 µg to 6, 250 µg/mL ^29^. However, pine oils are not as good antimicrobial agents as eugenol oil (MIC values between 2.5 to 5 µL/mL for most bacteria strains) not detected in the commercial pine oil tested in this study ^20^. As shown by Rivas da Silva (+)-α-pinene and (+)-β-pinene have good antimicrobial activity and are characterized by low toxicity to humans ^13^. On the other hand, the final effect depends on the type of microorganisms and tested strains. For example, *Staphylococcus aureu*s is considered to be more sensitive to essential oils than gram-negative *E. coli* ^30^. Although essential oils and their components have been studied for many years their mechanism of antimicrobial action remains unknown ^31,32^. In most cases, one or two compounds included in the oils play an essential role and reduce or hinder bacterial growth. The main mechanism of action refers to alterations in microbial cell membranes and structures, disruptions of transport systems or ion channels, very rapid reactions with ^·^OH radicals (from external and internal sources) and damage of receptors and enzymes ^31^.

The increasing interest in EOs as antibacterial agents and a range spectra of bacterial species that are sensitive to them resulted in a huge number of patents and applications ^32^. Unfortunately, already in 2008, it was noted that *S. aureus* adapted to the EOs of *M. alternifolia* after repeated use. For this reason to achieve satisfactory results (irreversible damage of bacterial cells) and avoid the evolution of resistance it is important to apply the EOs in high concentrations ^33^. However, such a procedure may be difficult to apply in practice. Essential oils undiluted essential cannot be applied directly to the skin. This follows from the fact that essential oils in high concentrations provoke skin, eye, nasal irritation, contact dermatitis or allergic reactions ^34^. Likewise in the food sector, only EOs with a strong antibacterial potential in very low concentrations (1% or below) are used ^35^. It means the novel ways for improving the antimicrobial performance of essential oil or microencapsulation, enabling protection and controlled release should be explored ^36^. The application of rotating magnetic field at varied frequency to increase efficacy of pine oil against *E. coli* was proposed in this study as one of possible alternatives. It was demonstrated that under electromagnetic field at a lower frequency (5 Hz) the tested oil had almost the same antimicrobial activity as in non-electromagnetic field conditions. Rapid reduction of bacterial number was observed in the case of RMF at the frequency range of 25–50 Hz in less than 100 min. The effect of rotating electromagnetic field on essential oil performance has not been reported yet. Upadhyaya and Patel showed that it was only a short-term rise in the antimicrobial potential of the *Jasminum grandiflorum* L. upon exposure of 12–24 h under Dipole Antenna transducer at 1800 MHz frequency, that revealed magnetic field strength of about 1.9 mG. It was concluded that exposure beyond 36 h resulted in a significant decline of antibacterial potential against selected pathogenic bacteria ^37^.

Further development of applications of electromagnetic field methods to increase the antimicrobial efficiency of antibacterial agents including antibiotics and nanoparticles could provide enormous potential to the developing new treatments ^38^. However, studies of dermatitis after contact with pine oils and RMF are needed. For further studies, it is proposed to examine other types of essential oils and other antibiotic-resistant bacteria (ARB) which are causing a serious concern throughout the world.

The new method can be applied in many fields e.g. aromatherapy, medicine as a component of dressing or as food preservative. From the technical perspective, the proposed method is profitable owing to the easy construction of the reactor or other external sources of the electromagnetic field. The application of electromagnetic field will allow to apply lower concentration of pine oil and can contribute to overcoming problems and limitations of essential oils such as intense aroma, reduced solubility, high reactivity, hydrophobicity and possible interactions with food ingredients or skin.

## Methods

All measurements were performed using an originally designed and constructed set-up (sketch and descriptions are presented in Supplementary Materials SM 5). The values of magnetic induction at different points inside the cylindrical process chamber were used according to the procedure of Rakoczy and Masiuk ^39^.

Based on the experimental results, the maximum and average values of the magnetic induction for the tested frequencies of the electrical current were obtained (Table 2).

**Table 2:** The maximum nd averaged values of the magnetic induction for the selected cross-sections of the RMF generator.

Frequency of electrical current	The maximum value of magnetic induction for the selected cross-section of the RMF* generator	The average value of magnetic induction for the selected cross-section of the RMF generator
5 Hz	25.36 mT	13.13 mT
25 Hz	37.06 mT	18.40 mT
50 Hz	42.64 mT	19.92 mT

*RMF—Rotating Magnetic Field.

Commercial *Pinus sylvestris* L. essential oil was purchased from a Polish company Avicenna-Oil® W. Podlaski (Wroclaw, Poland). The manufacturer description included: oil obtained from fresh needles and Scots pine twigs. The chemical composition of *Pinus sylvestris* essential oil was determined using a Hewlett Packard 6890 gas chromatography coupled with Hewlett Packard 5973 Mass Selective Detector operating at 70 eV mode. The essential oil sample (30 mg) was dissolved in dichloromethane (1.5 mL) and 1 µL of the solution was injected in a split mode at a ratio of 5:1. Chromatographic analysis of the essential oil sample was repeated three times. Component relative concentrations were calculated based on GC peak areas without using correction factors. The individual constituents were identified by comparison of their mass spectra with those stored in the Wiley NBS75K.L and NIST/EPA/NIH (2002 version) mass spectral libraries and confirmed by comparison of their retention indices (RI) with data reported in NIST Chemistry WebBook (https://webbook.nist.gov/chemistry/) The retention indices were calculated for all volatile constituents according to Van Den Dool and Kratz equation using a homologous series of n-alkanes (C7–C30, 1000 µL in hexane, Supel-co, Bellefonte, PA, USA) ^40^.

The reference strain of Gram-negative bacteria*, Escherichia coli* K12 (ATCC 25922 (*E. coli*) was tested. Bacteria were grown in Enrichment Broth (BIOCORP Sp. z.o.o., PL) at 37 °C for 24 h. The overnight cultures were transferred to sterile saline buffer (0.85% NaCl). Cultures were diluted until the final concentration of bacteria 0.5 in McFarland Standard (BioMerieux, PL) approx. 1.5 × 10^8^ (working suspension). In the beginning, the susceptibility of microorganisms towards pine oil disc diffusion tests was determined. In this experiment, antimicrobial activity of oil in concentration of 100% 50%, 25%, 12.5% and 6.25% was assayed with sterile paper discs Whitman paper no. 1 (Ø = 10 mm). Three oil-soaked discs (10 µL per disc) were placed on Petri dish plates with Plate Count Agar PCA (BioMaxima S.A., Poland). Control was made by dipping sterile paper discs into the sterile saline buffer. All plates were incubated at 37 °C for 24 h. After incubation, the inhibition zones around the disc were observed. This study was repeated twice to confirm the results.

In the next step minimum, the inhibitory concentration of oil by liquid dilution method was determined. The tested microorganisms were exposed to the two-fold serial concentrations of pine oil, ranging from 6.25 to 100% (v/v) and prepared in the same way as for the disc test. Then all diluted oils were assessed with viable counts procedure after 60, 120, 160 and 180 min of incubation at room temperature. The MIC was the lowest concentration of pine oil that prevented the visible growth of bacteria. To examine the effect of pine essential oil and rotating magnetic field RMF) on antibacterial performance four dilutions of oil in sterile saline buffer (0.85% NaCl, Chempur Poland) were prepared. Suspensions in concentrations: 100%, 50%, 25%, 12.5% and 6.25% (prepared according to the above mentioned procedure) with addition 0.02% of surfactant Tween™ 80 were investigated. Then 0.15 mL of working suspension of bacteria was added to 3 mL of glass test tubes containing each prepared suspension of oil and control tube containing only 3 mL of saline buffer. All tubes prepared in duplicate were placed in two reactors (6 under magnetic field and 6 without magnetic field). Both reactors were thermostated to maintain the temperature at 37 °C . The samples were taken every 20 min. A series of decimal dilutions were made (0.5 mL of working dilution with 4.5 mL of NaCl). 0.25 mL diluted solutions were placed on Standard Plate Count Agar. The experiment was continued for 160 min. In the same way, the bacterial number at the “0” was determined. All the plates were incubated at 37 °C for 24 h. Viable colonies on the plates were counted and converted to the average logarithm of colony-forming units per milliliter (CFU/mL).

## Supplementary Information

Supplementary Information.
