# Supplementary material for: Effect of pine essential oil and rotating magnetic field on antimicrobial performance
Source: Sci Rep. 2022 Jun 11;12:9712. doi: 10.1038/s41598-022-13908-5 (PMC9188566; doi:10.1038/s41598-022-13908-5)
Supplement: Supplementary file 1 — Supplementary Information. [file 41598_2022_13908_MOESM1_ESM.docx]

**SM1 .** Chemical composition of pine essential oil.

| No. | Compounds | Class | R_t_ [min] | RI_Exp._ | RI_Lit._ | % |
| --- | --- | --- | --- | --- | --- | --- |
| 1. | 2-Heptanone | O | 8.04 | 884 | 886 | 0.25 |
| 2. | α-Tricyclene | MH | 8.96 | 920 | 921 | 0.19 |
| 3. | α-Thujene | MH | 9.11 | 926 | 925 | 0.06 |
| 4. | α-Pinene | MH | 9.30 | 934 | 934 | 28.58 |
| 5. | Camphene | MH | 9.64 | 947 | 947 | 1.86 |
| 6. | Thuja-2,4(10)-diene | MH | 9.79 | 953 | 954 | 0.26 |
| 7. | Sabinene | MH | 10.22 | 970 | 970 | 0.13 |
| 8. | β-Pinene | MH | 10.39 | 976 | 976 | 17.79 |
| 9. | β-Myrcene | MH | 10.73 | 990 | 991 | 2.60 |
| 10. | δ-2-Carene | MH | 10.93 | 998 | 998 | 0.18 |
| 11. | α-Phellandrene | MH | 11.07 | 1003 | 1003 | 0.46 |
| 12. | δ-3-Carene | MH | 11.24 | 1010 | 1010 | 14.17 |
| 13. | α-Terpinene | MH | 11.38 | 1015 | 1015 | 0.87 |
| 14. | p-Cymene | MH | 11.58 | 1023 | 1024 | 0.78 |
| 15. | Limonene | MH | 11.71 | 1028 | 1028 | 11.58 |
| 16. | (Z)-β-Ocimene | MH | 11.91 | 1036 | 1037 | 0.46 |
| 17. | γ-Terpinene | MH | 12.45 | 1058 | 1059 | 0.21 |
| 18. | Terpinolene | MH | 13.20 | 1087 | 1088 | 4.62 |
| 19. | Linalool | OM | 13.48 | 1098 | 1098 | 0.08 |
| 20. | Fenchol | OM | 13.85 | 1114 | 1115 | 0.12 |
| 21. | α-Campholenal | OM | 14.19 | 1127 | 1127 | 0.05 |
| 22. | (E)-p-Menth-2-en-1-ol | OM | 14.39 | 1136 | 1135 | 0.08 |
| 23. | *trans*-Pinocarveol | OM | 14.49 | 1140 | 1140 | 0.10 |
| 24. | Camphor | OM | 14.63 | 1145 | 1145 | 0.15 |
| 25. | Borneol | OM | 15.14 | 1166 | 1166 | 0.18 |
| 26. | Terpinen-4-ol | OM | 15.41 | 1177 | 1177 | 0.09 |
| 27. | α-Terpineol | OM | 15.72 | 1190 | 1190 | 0.88 |
| 28. | Myrtenal | OM | 15.89 | 1197 | 1197 | 0.11 |
| 29. | Bornyl acetate | OM | 17.96 | 1286 | 1287 | 4.55 |
| 30. | α-Cubebene | SH | 19.41 | 1351 | 1351 | 0.12 |
| 31. | α-Longipinene | SH | 19.47 | 1354 | 1356 | 0.13 |
| 32. | Ylangene | SH | 19.93 | 1375 | 1374 | 0.05 |
| 33. | α-Copaene | SH | 20.01 | 1379 | 1378 | 0.29 |
| 34. | β-Elemene | SH | 20.34 | 1394 | 1394 | 0.05 |
| 35. | Longifolene | SH | 20.71 | 1411 | 1412 | 0.98 |
| 36. | Caryophyllene | SH | 20.99 | 1424 | 1423 | 5.18 |
| 37. | β-Copaene | SH | 21.15 | 1432 | 1433 | 0.05 |
| 38. | (E)-β-Farnesene | SH | 21.60 | 1454 | 1455 | 0.06 |
| 39. | α-Caryophyllene | SH | 21.70 | 1458 | 1459 | 0.75 |
| 40. | 1-Dodecanol | O | 22.07 | 1476 | 1478 | 0.06 |
| 41. | Bicyclogermacrene | SH | 22.61 | 1502 | 1505 | 0.06 |
| 42. | β-Bisabolene | SH | 22.74 | 1509 | 1508 | 0.07 |
| 43. | δ-Cadinene | SH | 23.07 | 1525 | 1525 | 0.29 |
| 44. | Caryophyllene oxide | OS | 24.33 | 1589 | 1589 | 0.29 |
|  | Total |  |  |  |  | 99.87 |

RIExp. – Retention indices relative to n-alkanes (C7-C30) on a HP-5 MS capillary column

RILit. – Literature retention indices (NIST Chemistry WebBook: https://webbook.nist.gov/chemistry/)

**SM2.** GC-MS chromatogram of pine essential oil


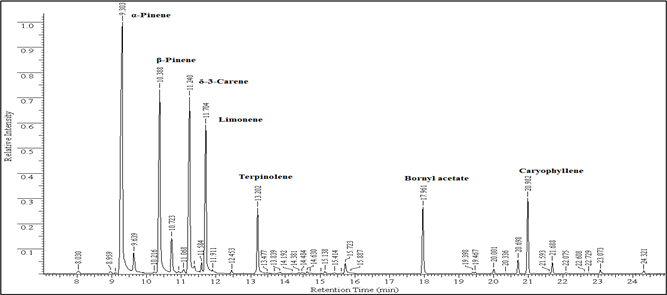


| **Concentration [%]** | **Means ± S.D of zones of inhibition by essential oils [mm]** | |
| --- | --- | --- |
| 100.0 % | | 10.0 ± 1.4 |
| 50.0% | | 1.5 ± 1.4 |
| 25.0% | | 0.0 ± 0.0 |
| 12.5 | | 0.0 ± 0.0 |
| 6.25% | | 0.0 ± 0.0 |
| Control | | 0.0 ± 0.0 |

**SM3.** Pine essential oil zones of inhibition against *E. coli.*

**SM4. The values of parameters of Eq. (2) and the coefficients of determination (R2) for the response surface graphs (Figures 3-6).**

| Parameter of Eq. (2) | Control  (Figure 3) | f = 5 Hz  (Figure 4) | f = 25 Hz  (Figure 5) | f= 50 Hz  (Figure 6) |
| --- | --- | --- | --- | --- |
| p_1_ | 0.96663 | 0.98310 | 1.26049 | 0.97771 |
| p_2_ | 0.00113 | 0.00536 | 0.00247 | 0.00267 |
| p_3_ | 0.00001 | 0.00002 | 0.00001 | 0.00002 |
| p_4_ | 0.00053 | 0.00076 | 0.02205 | 0.00661 |
| p_5_ | 0.00002 | 0.00001 | 0.00025 | 0.00021 |
| p_6_ | 0.00001 | 0.00007 | 0.00005 | 0.00009 |
| R_2_ | 0.84001 | 0.94199 | 0.84722 | 0.91018 |

**SM.5 Sketch and description of experimental set-up**


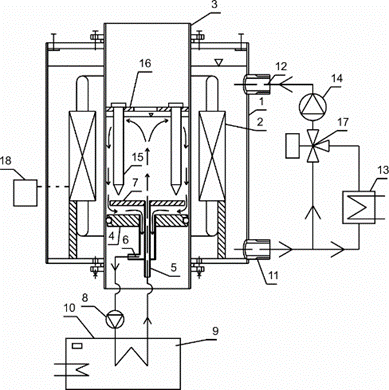


Sketch of experimental set-up: 1 – housing; 2 – generator of RMF; 3 – cylindrical conduct; 4 – bottom; 5 – reactor chamber inlet; 6 – reactor chamber outlet; 7 – baffle; 8 – circulating pump; 9 – coil; 10 – thermostat; 11 – tank outlet; 12 – tank inlet; 13 – heat exchanger; 14 – circulating pump; 15 – probe; 16 – probe rack; 17 – valve with regulator; 18 – a.c. transistorized inverter.

The magnetically assisted reactor (MAR) consists of the housing (1) and the generator of RMF (2). The cylindrical conduct (3) was axially aligned with the RMF generator (2). The RMF was generated using the stator of a three-phase squirrel cage induction motor. This stator was connected to the alternating current (a.c.) transistorized inverter (18). The applied apparatus allowed to adjust the frequency of the electrical current (f) in the range between 5 and 50 Hz. The cylindrical conduct (3) has a bottom (4) in which a pipe-in-pipe inlet (5) and an outlet (6) of the reactor chamber is mounted. The cylindrical baffle (7) is mounted above the bottom (4). The diameter of this baffle is smaller than the internal diameter of the cylindrical conduct (3). The gap between the conduct and the baffle allows the flow of the liquid to the thermostat of the process chamber. In case of these investigations, the demineralization water was used to keep the stable temperature of probes in the RMF generator (2). The outlet (6) is connected to the inlet (5) by the coil (9) that is placed in the thermostat (10). The circulating pump (8) is used to flow the liquid through the coil (9). Moreover, the RMF generator windings were liquid-cooled using an external heat collection system. The housing (1) is equipped with an inlet (11) and an outlet (12) for liquid coolant. The silicone oil flowing through the reactor absorbed the excess heat from the windings. This heat was removed in the heat exchanger (13). The circulation pump (14) was circulating the oil between the reactor and the heat exchanger (13). The value with regulator (17) was used to monitor the temperature of silicone oil flowing into the reactor. The applied system of the temperature stabilization allowed to keep the constant temperature inside the reactor chamber at 37 ± 0.5 ⁰C. The probes (15) with the rack (16) were placed inside the RMF generator (2). To stabilize the temperature inside the reactor's process chamber, an additional temperature control system was used. The probes (9) were placed in a water bath at 37℃. The circulation pump (10) forced the flow of water between the chamber of the reactor and the heat exchanger (11). To establish the stable temperature of the liquid inside the rector (37 ± 0.1 ⁰C), the working liquid (water circulating between the reactor and the heat exchanger) was heated in the countercurrent heat exchanger (11) by the water from the thermostat (12).

The obtained values of magnetic induction at different points inside the RMF generator are presented in the form of patterns of magnetic induction. **Fig 1** shows the contour patterns of the spatial distributions of the magnetic field in the selected cross-section of the RMF generator. It should be noticed that the presented patterns of the magnetic induction were obtained for the frequencies of the electrical current equal to 5, 25 and 50 Hz. The experimental procedure was carried out by using these frequencies of the electrical current. Based on the investigations of the magnetic in-duction, the maximal values of this parameter, B_max_, might be obtained. It should be noticed that the maximum values of magnetic induction are in the area near the RMF generator. The values of the magnetic induction are decreased towards the centre of the RMF generator. As follows from the analysis of the experimental data, the values of magnetic induction are spatially distributed in the volume of the RMF generator. Therefore, the applied RMF might be characterized employing the averaged values of magnetic induction.


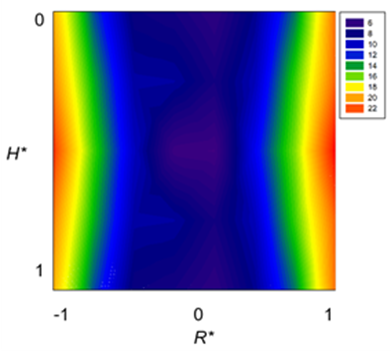

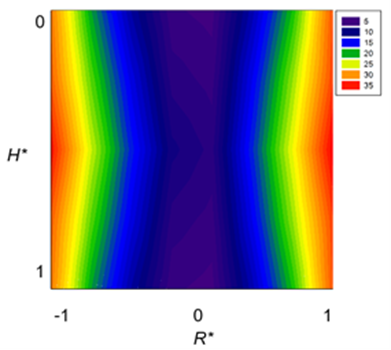


1. **(b)**


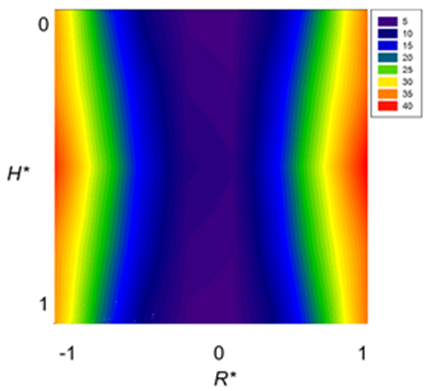


**(c)**

The contour pattern of the spatial distribution of the magnetic induction in the selected cross-section of the magnetically assisted photoreactor (MAP) for the frequency of the electrical current equal to: **(a)** 5 Hz; **(b)** 25 Hz and **(c)** 50 Hz.
